# Diverticular Bleeding: A Clinical Image

**DOI:** 10.7759/cureus.18350

**Published:** 2021-09-28

**Authors:** Christopher F Brewer, Yayha Al Abed

**Affiliations:** 1 Plastic Surgery, Addenbrooke's Hospital, Cambridge University Hospitals NHS Foundation Trust, Cambridge, GBR; 2 General Surgery, Broomfield Hospital, Chelmsford, GBR

**Keywords:** diverticulosis, clinical image, colonoscopy, pr bleeding, diverticulum

## Abstract

A man in his 40’s was admitted to the general surgery ward with multiple episodes of large amounts of painless bright red per rectal (PR) bleeding and passage of clots. Urgent outpatient colonoscopy revealed a diverticulum which was associated with a wide diameter blood vessel originating from its base which was not actively bleeding. The clinical picture presented by the colonoscopy is one of the first to clearly identify large caliber blood vessels emerging from a colonic diverticulum.

## Introduction

Diverticular disease is a relatively common condition, estimated to affect 50% of the population over the age of 60 years [1]. The diverticula often form around blood vessels that penetrate the muscular layers of the bowel, predisposing pockets of mucosa to herniation, obstruction, and infection. The juxtaposed vasculature can subsequently rupture, precipitating per rectal bleeding. Indeed, previous studies have suggested that over 26% of all lower gastrointestinal bleeding episodes in the United Kingdom are secondary to diverticular disease [2].

Despite its frequency, there have been very few clinical images or media which capture the close relationship of the diverticula and blood vessels. In this report, we discuss the case of a man who presented with suspected diverticular bleeding and shows a colonoscopy image that highlights the juxtaposition of intestinal vasculature and a diverticulum.

## Case presentation

**Figure 1:**
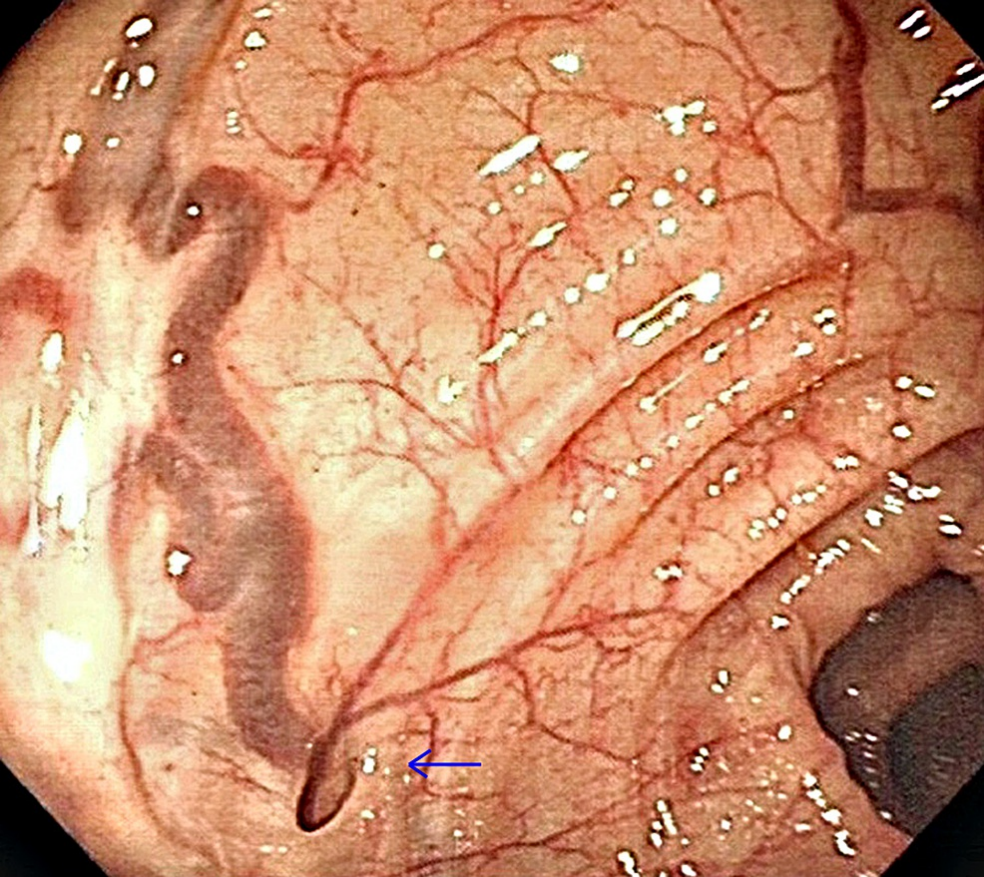
Diverticulum with juxtaposed blood vessels identified on colonoscopy (blue arrow) Diverticulum with juxtaposed blood vessels identified on colonoscopy.

A man in his 40’s was admitted to the general surgery ward with multiple episodes of large amounts of painless bright red per rectal (PR) bleeding and passage of clots. Fresh blood was identified on PR examination, however, there was no evidence of anorectal pathology. Proctoscopy did not reveal evidence of hemorrhoids. Hemoglobin on admission was 95g/dL (baseline 128g/dL). The patient was conservatively managed and discharged on day two with no further episodes of bleeding. An urgent outpatient colonoscopy revealed diverticula within the wall of the sigmoid colon. One such diverticulum was associated with a wide diameter blood vessel originating from its base which was not actively bleeding (Figure 1). No endoscopic intervention was required. The patient was discharged the same day following dietary and lifestyle advice and has remained symptom-free. In this case, we believe that bleeding originated from diverticular disease.

## Discussion

Diverticular disease is a relatively common condition that can often present with PR bleeding. In this report, we present the case of a young man who was briefly admitted to the general surgery ward with a suspected diverticular bleed which resolved spontaneously. Indeed, it is estimated that hemorrhage resolves spontaneously in approximately 80% of these patients, with no surgical intervention required [3]. However, up to 30% will have an additional episode of bleeding [4].

The current guidance in the United Kingdom is that patients are risk-stratified on admission to hospital according to the severity of the bleed and likelihood of adverse outcomes [5]. Patients classified as low risk may be discharged with urgent outpatient colonoscopy.

Figure 1 shows the subsequent outpatient colonoscopy image for this patient demonstrating a large caliber blood vessel juxtaposed to a colonic diverticulum. While there have been several detailed clinical images of colonic diverticula [6-8] and bleeding diverticula published, few have clearly illustrated the close relationship with perforating vasculature [9]. Young-Fadok et al. published clinical images of colonoscopy findings from a 57-year-old man who presented with micro-perforations of colonic diverticula [10]. While their images do show juxtaposed vasculature, the vessel caliber is considerably smaller relative to the diverticular size in our clinical image, and the patient did not present with lower GI bleeding. Similarly, Haji et al. demonstrate several colonoscopy images from patients with diverticular disease with only small blood vessels emerging from each diverticulum [11].

The vasculature shown in our clinical image is of significant caliber relative to the diverticulum and appears to project off the surface of the bowel mucosa. This may predispose the luminal surface to erosions from bowel contents. Further, vascular intimal weakening may also occur due to the inflammatory effects from intermittent episodes of diverticulitis, subsequently leading to lower GI bleeds.

While it is not possible to definitively say whether this was the source of bleeding, no other identifiable sources were found on colonoscopy. Indeed, one of the drawbacks of outpatient colonoscopy is the decrease in diagnostic and potential therapeutic yield [12]. Nevertheless, previous reports have demonstrated no significant difference in mortality, length of stay or costs with early colonoscopy for diverticular disease [13].

## Conclusions

The clinical picture presented by the colonoscopy is one of the few published reports showing large caliber blood vessels emerging from a colonic diverticulum, which may have been the cause of a lower GI bleed. The majority of these cases can be managed non-surgically, however, outpatient colonoscopy is recommended to identify pathology and potential source of bleeding.
